# One Eye Open: Reflections on Embedding Lived Experience in Sleep, Circadian Rhythms, and Mood Disorder Research

**DOI:** 10.1111/hex.70614

**Published:** 2026-03-09

**Authors:** Samuel J. Hockey, Sarah McKenna, Alexander Hill, Andrew W. Shim, Annabel Ramsay, Nayonika D. Bhattacharya, Tara Lindsay‐Smith, Nathan Bradshaw, Elie Jeon, Yun Ju Song, Jacob J. Crouse, Ian B. Hickie

**Affiliations:** ^1^ Youth Mental Health & Technology Team, The Brain and Mind Centre The University of Sydney Sydney New South Wales Australia

**Keywords:** circadian rhythm, co‐production, lived experience, mood disorders, sleep, young people

## Abstract

**Background:**

The integration of lived experience within mental health research remains inconsistent, with many models positioning lived experience in advisory rather than embedded roles. This can limit meaningful engagement and reduce the relevance and translational impact of research.

**Approach:**

Drawing on an ongoing five‐year Welcome Trust‐funded programme investigating sleep and circadian rhythm disturbances in youth‐onset mood disorders, this viewpoint reflects the development and function of a Lived Experience Working Group embedded within the Brain and Mind Centre's Youth Mental Health & Technology Team. Using this programme as a case study, we examine how co‐production is implemented across research design, governance, interpretation of findings, and knowledge dissemination.

**Key Insights:**

We outline how co‐production is actioned and refined across the lifecycle of a complex research programme. We highlight lived experience contributions alongside relational and structural tensions, and practical challenges relating to inclusion, power‐sharing, scalability, and translation.

**Conclusion:**

Embedding lived experience as a core form of expertise requires intentional governance structures, reflexive practices, and sustained institutional investment. This article offers practical and reflective guidance for researchers seeking to meaningfully integrate lived experience within youth mental health research.

## 1 Experience as Expertise

‘I don't want data to be captured just around poor sleep, when it might be because of a mood disorder and not poor sleep practices. It would be incredible to understand the information related to sleep, particularly what I can do to better my sleep if I also have a mood disorder.’

Annabel Ramsay,

Lived Experience Working Group member

### 1.1 Embedding Lived Expertise

**Figure 1:**
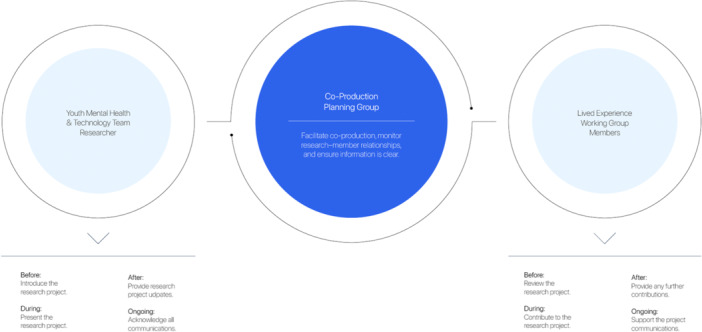
Roles and responsibilities across the co‐production research cycle (2024).

Historically, research has divided academics and research participants, often creating an ‘us versus them’ mentality [1]. This separation may contribute to the almost 20‐year gap between clinical trials and real‐world use of mental health interventions [2]. Within the Brain and Mind Centre's BMC Youth Mental Health & Technology (YMHT) Team, young people with lived experience are not just participants but are equal partners shaping research priorities, design, and interpretation [3, 4]. Through co‐production, embedding of lived experience occurs at every stage of the research lifecycle, enabling real‐time insights that support more practical, informed, and responsive mental health solutions [3, 4]. Accordingly, we embed young people with lived experience as partners through iterative co‐design and feedback processes, strengthening trust, relevance, and research quality (Figure 1) [5, 6].

The goal of this paper is to provide a reflexive account of how co‐production with lived experience is embedded, governed, and sustained across a multi‐year youth mental health research programme. Rather than reporting empirical outcomes, this viewpoint prioritises reflection on co‐production processes, relational infrastructures, and decision‐making practices [4, 5]. In doing so, this paper offers methodological and experience‐based insights intended to support other researchers seeking to meaningfully embed lived experience and co‐production within mental health research.

### 1.2 Inclusive Recruitment

Early recruitment experiences highlighted structural barriers such as stigma, inequity, and academic prejudices; recruitment processes were therefore designed to prioritise relational engagement, transparency, and flexibility. Recruitment to the Lived Experience Working Group (LEWG) was intentionally designed to support intersectionality and meaningful participation. Targeted outreach through youth mental health research networks, clinical mental health services, and community organisations (e.g., Multicultural Youth Advocacy Network Australia, Batyr, and headspace) sought to engage young people typically under‐represented in academic research [3, 4].

Recruitment prioritised diversity across culture and language, gender and sexuality, disability, socioeconomic background, and experiences of mental ill‐health and care pathways. Materials were co‐developed with lived experience researchers to ensure accessible language, clear expectations, and transparency regarding involvement, aligning with co‐design principles that emphasise accessibility and shared ownership [5]. Eligibility was based on lived experience expertise rather than formal qualifications, reinforcing experiential knowledge as central to the group's function [3, 4]. Informed choice was emphasised through an interview prior to joining, outlining roles, time commitment, and flexible participation, supporting individuals' capabilities and interests (as reflected in the LEWG's Terms of Reference) [4].

Flexible engagement was intentionally designed to support participation across communication preferences, cultural norms, and changes in circumstances [3, 4]. This flexibility supports responsiveness to diversity while maintaining relational trust over time. Co‐production within this programme has been implemented as a continuous, iterative cycle across five domains: co‐discovery, co‐design, co‐evaluation, co‐planning, and co‐delivery, each embedding lived experience and shared decision‐making across all stages of the research programme (Figure 1; Panel 1).

### 1.3 Meaningful Research Impact

In June 2024, a 5‐year Wellcome Mental Health Award was launched to investigate Sleep and Circadian Rhythm Disturbances (SCRDs) and mood disorders in young people [4, 5]. As mentioned, this research programme integrates co‐production across all stages of the research, aligning with Wellcome Trust's commitment to lived experience–driven mental health research (Panel 1) [4, 5]. The influence of co‐production within this programme is most clearly reflected in how young people articulate the personal and practical value of the research. LEWG members described SCRDs as part of a complex ‘cocktail of symptoms’, making it difficult to distinguish causes and consequences. Being able to identify how specific symptoms relate to identifiable variables was described as ‘empowering’, particularly in reducing fear and uncertainty and supporting more informed self‐management.

**Figure 2:**
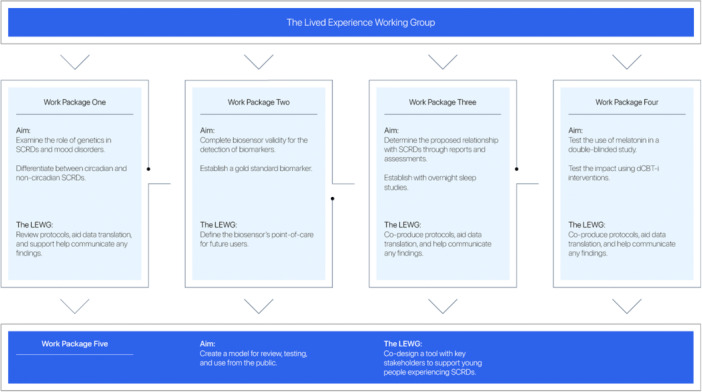
Overview of the five Work Packages.

This approach aligns with broader efforts to address power‐sharing, complexity, and the risks of tokenism in mental health research, while emphasising the need for ongoing critical reflection [1, 2, 6]. The programme is comprised of five interconnected Work Packages investigating key factors influencing SCRDs and mood disorders in young people, each shaped through co‐production with the LEWG and grounded in lived expertise (Figure 2).

Panel 1 **Shaping through Co‐production**

**Table:** 

1. *Co‐Discover* Using novel biosensor technologies, we are exploring new ways to measure biomarkers related to SCRDs, track circadian rhythms over time, and assess responses to sleep and circadian interventions (e.g., exogenous melatonin). The LEWG identified both the need and interest in measuring circadian rhythms beyond traditional in‐lab settings and the value of these insights in informing more personalised, early intervention strategies. 2. *Co‐Evaluate* Through self‐reported and clinical assessments, overnight sleep studies, and wearable devices (e.g., actigraphy watches, light sensors), we track sleep‐wake patterns and environmental factors influencing circadian rhythms. LEWG members provide feedback on user experience, including barriers to participation, to enhance accessibility and inclusivity, while accounting for cultural, geographic, and socio‐economic considerations. 3. *Co‐Design* LEWG members, researchers, and clinicians work together to co‐design study methodologies, including protocols, recruitment strategies, and trial branding, ensuring study design remains relevant to diverse lived experiences. This process ensures our research extends beyond quantitative outcomes to reflect young people's immediate needs, rather than being focused solely on quantitative data. 4. *Co‐Plan* During planning for overnight monitoring studies, LEWG members share feedback about their own participation experiences, including negative experiences and areas for improvement. Their lived expertise has informed procedural changes from early planning stages, supporting participant comfort and clearer communication of study expectations (e.g., biological sampling, sleep study environment). 5. *Co‐Deliver* Formal co‐authorship and involvement in dissemination activities reinforce shared power and participation while prioritising ongoing collaborative relationships and capability‐building opportunities. Transparent acknowledgement helps mitigate risks of tokenism, which remains a real threat to the integrity of co‐produced research. As reflected in this paper's authorship, LEWG members have opportunities to co‐author on project outputs and contribute directly to knowledge translation.

## 2 From Insight to Action

‘If I could have some sort of biosensor test, like a diabetes thing, that could just help me understand what's going on, the physiological stuff and all of that, and be more educated about my cycling through moods, that would be life changing for me. Absolutely life changing. And if I could have ways of shortening and making episodes safer, also life changing.’

Anna Treneman,

Lived Experience Working Group member

### 2.1 Co‐Production in Practice

We implemented co‐production through ongoing collaboration with the LEWG, shaping research priorities, methods, and translation across the programme (Figure 2). Members described how accessing a biosensor‐style tool could help them ‘understand what's going on’ physiologically when ‘cycling through moods’, an insight described as potentially ‘life changing’ in supporting safer, more informed self‐management (Panel 1).

### 2.2 Changes Driven by Co‐Production

Building on these insights, co‐production was used as a structured process for ongoing dialogue between researchers and LEWG, rather than isolated consultations. Through reflective discussions, this process spans across the programme's protocol revisions, defining point‐of‐care and data translation, enabling lived expertise to be translated into design decisions, governance practices, and trial procedures [7]. For example, members reflected that conceptualising SCRD‐related biomarkers as a familiar self‐monitoring tool could support ongoing self‐monitoring and agency, rather than passive data collection.

Formal recognition through co‐authorship and dissemination, as well as shared responsibilities and feedback mechanisms, reinforced value and supported capability‐building, contrasting prior experiences of informal or unacknowledged involvement and demonstrating how shared decision‐making redistributes power within the research process (Figure 1; Panel 1).

During the co‐evaluation and co‐design phases of the MELODY Sleep Trial, LEWG contributions were integrated through regular meetings and shared review of draft protocols, informing refinements that may otherwise have been overlooked, including changes to feedback mechanisms, compensation considerations, and equity‐focused design decisions (Panel 1) [7].

## 3 Safeguarding Co‐Production

‘This research will be helpful in providing a degree of certainty. Like, there are lots of tangible ways that this could be useful, but I think that when there's a cocktail of symptoms I'm experiencing, and they're all coming in from unknown areas, it's very easy to bundle them together in a scary way. And so, I think that if I can say, “Ah yes! Symptom ‘x’ is being produced by variable ‘y’ that I can see this,” I know that will be an empowering piece of information to have access to.’

Alexander Hill,

Lived Experience Working Group member

### 3.1 Lived Experience Leadership

Lived experience leadership strengthened both governance and psychosocial safety. LEWG members have emphasised that being surrounded by others who have ‘also experienced this stuff’ contributes to a sense of safety when engaging with more vulnerable experiences in research, highlighting lived experience leadership as both a governance and psychosocial safeguard (Figure 1).

Within this research programme, we embedded safeguarding processes, including pre‐ and de‐briefings, flexible modes of contribution (i.e., verbal, written, or anonymised), alongside the appointment of a Lived Experience Researcher as a Chief Investigator (Panel 1). This role provides structural accountability for co‐production, ensuring lived expertise is consistently understood, respected, and formally acknowledged across each stage of this programme, in line with the YMHT Team's published protocol for iterative co‐production (Figure 1) [4]. These contributions are documented through structured feedback loops, the use of plain‐language materials (supporting shared understanding), and clearly establishing expectations regarding roles, remit, and decision‐making (Figure 1). Together, these processes, supported by a reflexivity monitoring and evaluation, help young people with lived experience be equal partners in shaping our research [4].

### 3.2 Safety and Capability

To ensure our members feel safe and supported throughout the research programme, our modes of co‐production must continually be evaluated and improved where necessary. We've implemented formal processes to increase agency and comfort in members, such as a structured feedback‐loop co‐designed with LEWG members, pre‐briefings for readiness, and debriefs with co‐facilitators to support psychological safety (Figure 1). Still, members often find critiquing academic research challenging due to barriers, such as structural and perceived power imbalances within academic research through formal and informal feedback channels. We recognise the leadership role of the Lived Experience Researcher as critical in supporting members to navigate these challenges, while meaningful co‐production also requires researchers to share decision‐making power, engage where possible, and invest in relationships and capability‐building.

## 4 Discussion

‘Remedying central tokenisation is frustrating because the onus is put on us to fix it. And if you're the only one who has to constantly speak up about a particular issue, you're going to feel like a token, isolated, and shit half the time. It's unsustainable and not reliable.’

Nayonika Bhattacharya,

Lived Experience Working Group member

### 4.1 Shared Responsibility for Inclusion

Genuine investment in co‐production must centre the inclusion of ethnically and culturally diverse young people, whose lived experiences strengthen the relevance of research. However, meaningful engagement requires structural flexibility and responsiveness. For example, LEWG members highlighted that participation in clinical studies such as the MELODY Sleep Trial can be affected by sleep disruptions linked to cultural and religious expectations, a factor often overlooked in traditional research planning [3, 7]. Similarly, monthly LEWG meetings may conflict with religious calendars, underscoring the need to reconfigure engagement processes without compromising involvement. In response, we introduced flexible modes of contribution (e.g., asynchronous feedback and online platforms) and accommodated variability in attendance.

While the inclusion of diverse lived experience voices enriches research, several members expressed initial hesitancy due to past experiences of tokenism [1, 3, 4, 5]. Through intentional recruitment of marginalised and minoritised participants, we sought to protect emotional safety and reduce the burden of being ‘the only one’ from a community and the associated pressure to ‘constantly speak up’ [4]. These tensions highlight inclusion as a shared ethical responsibility that requires ongoing attentiveness, relational investment, and institutional support.

### 4.2 Limitations of This Approach

While this approach to co‐production offers a promising pathway for embedding lived experience in mental health research, it is not without limitations. Sustaining meaningful co‐production requires substantial investment in time, relational and interpersonal dynamics, and funding [3, 4, 5]. The approach described in this paper is supported by multi‐year grant funding; similar resourcing is unlikely to be available across many research contexts. Recruiting and retaining a diverse group of young people with lived experience also remains challenging, particularly where fluctuating mental health, competing responsibilities, and broader structural inequities influence participation [1, 3]. Despite intentional recruitment strategies, engagement inevitably varies over time.

This paper's approach relies on clear governance structures and lived experience leadership roles. Where such roles are unable to be institutionally supported, the fair redistribution of power across lived experience and research domains may prove difficult to achieve [4, 5, 6]. Importantly, this paper reflects one programme‐specific approach, with limitations related to generalisability, scalability, and sustainability [5, 6]. As such, similar approaches will require deliberate reflexivity and ongoing adaptation rather than static replication.

### 4.3 Generalisation Versus Personalisation

Beyond the structural limitations outlined above, co‐production surfaced challenges in how sleep and circadian research are typically translated into practice. Interventions to improve sleep and circadian rhythms are often generic behavioural and/or routine‐based changes, relying on blanket recommendations that fail to account for individual biological variation, comorbidity (mental and physical), and lived context [3, 4]. Such approaches place the onus on the individual and frame sleep difficulties as behavioural rather than relational or contextual. LEWG members described frustration with the common sleep hygiene recommendations that overlook complex and fluctuating symptom experiences.

LEWG members also noted a disconnect between knowing what is recommended and meaningful application. When experiences are layered and unpredictable, generic advice limits agency, with attempts to adhere to generic recommendations described by the LEWG as anxiety‐provoking when individuals ‘can't even get up’. This highlights a broader tension for current knowledge translation approaches that prioritise generalisation over personalisation [1, 6].

Importantly, LEWG members emphasised that effective translation requires contextualisation. Being able to ‘understand what's going on’ physiologically and distinguish whether changes were driven by mood or behaviour was described by the LEWG as empowering, particularly for early intervention and self‐management. Members cautioned that overly technical or ‘alphabet soup’ biomarkers risk overwhelming users, reinforcing the importance of co‐designing communication strategies that are clear and acceptable to end users (Panel 1) [5]. These reflections informed the programme's emphasis on pairing biosensor‐derived data with co‐produced interpretation, supporting personalised and actionable understanding while remaining attentive to accessibility and burden [2, 5].

## 5 Conclusion

‘If I clearly see the research and I engage with it, and it's really laid out for me the cause and benefit and I understand the importance of it a bit better, maybe that would help with making better choices.’

Tara Lindsay‐Smith,

Lived Experience Working Group member

### 5.1 Learning Through Co‐Production

In this paper, we reflect on the process of embedding lived experience through co‐production, integrating methodological and lived experience insight. Our reflections demonstrate how co‐production has been, and continues to be, implemented, challenged, and refined across a multi‐year research programme focused on the relationship between SCRDs and mood disorders in young people (Figure 2; Panel 1).

Across the programme, LEWG members consistently emphasised the importance of transparent, accessible, and practical research when navigating complex symptom experiences. When research processes and aims were clearly ‘laid out’, members described greater understanding of its ‘cause and benefit’, fostering engagement that felt meaningful and respected. This clarity supported agency, enabling more informed contributions and ‘better choices’ grounded in lived experience rather than generalised assumptions. Through this co‐production, three interrelated lessons emerged: (i) accessible and inclusive communication; (ii) flexible modes of contribution; and (iii) recognising the emotional burden on marginalised and minoritised individuals.

Clear communication is foundational to meaningful involvement across research participation and dissemination (Panel 1) [3, 4]. Therefore, flexibility must be intentionally designed from the outset while remaining responsive to contexts and the shifting needs shifting over time [3, 4]. Designing for variability in time commitments, communication modes, and member capabilities supports more equitable participation and helps reduce unintended exclusion or bias [5]. Finally, emotional and relational labour must be actively recognised and minimised; without this, co‐production risks tokenism, rather than shared decision‐making or contextually competent research [5, 6]. Not attending to these issues was described by a LEWG member as ‘unsustainable and not reliable’. Together, these lessons reinforce co‐production as an evolving practice requiring continual reflection and accountability (Figure 1) [4, 5].

### 5.2 Taking Lessons Forward

For this research programme, embedding lived experience through the LEWG reshaped both how research was conducted and what was prioritised across each stage. Co‐production informed study design, protocol development, participant materials, translation, and dissemination across all five Work Packages (Figure 2; Panel 1) [3, 4]. Reflecting across these processes, several transferable applications emerged that may be relevant for researchers working with lived experience groups (Panel 2).

Going forward, these applications highlight the importance of continued investment in lived experience leadership, reflexive governance, and transparent communication. Rather than offering a prescriptive model, they position co‐production as an evolving practice that benefits from ongoing reflection, adaptation, and documentation, resulting in intuitive research outcomes (Panel 2) [6]. Within this programme, embedding lived experience across all Work Packages aims to strengthen the relevance, integrity, and real‐world impact of research developed alongside young people experiencing SCRDs and mood disorders.

Panel 2 **Key Applications for Embedding Lived Experience Through Co‐Production**

**Table:** 

1. *Embedded Co‐Production Across the Research Cycle* Co‐production was most effective when embedded across the entire research lifecycle, rather than confined to isolated consultations [4, 5]. In this programme, lived experience input shaped discovery, design, evaluation, planning, and delivery, enabling insights to influence research questions, methods, and translation in real time. 2. *Investing in Relational and Governance Infrastructure* Relational and governance infrastructure is critical. Clearly defined roles, shared decision‐making, and formal recognition of lived experience leadership facilitated trust, accountability, and sustained engagement. In turn, this helps ensure lived experience is not only consulted but expertise is meaningfully integrated into all research processes and outputs [5, 6]. 3. *Design for Accessibility, Flexibility, and Emotional Labour* Continuous attention to accessibility, flexibility, and emotional labour is paramount. Providing flexible modes of contribution options, using plain‐language materials, and acknowledging relational and interpersonal dynamics helps to reduce burden and avoid tokenism. These practices support participation across fluctuating capabilities and intersectional contexts.

## Author Contributions

The first author, S.J.H. was responsible for the conceptualization and design of the article, interpretation and analysis of material informing the viewpoint, and drafting of the manuscript. Co‐authors Y.J.S., J.C., and I.B.H. contributed to the conceptual development of the article and refinement of its framing and overall position. S.M. provided supervision and contributed to critical review of the manuscript. Programme coordinators N.B. and E.J. contributed to manuscript review and feedback. Lived Experience Working Group members A.H., A.S., A.R., N.D.B., and T.L.S. contributed lived experience perspectives and manuscript review and feedback. All authors contributed to the critical revision of the manuscript and approved final version.

## Conflicts of Interest

The authors declare no conflicts of interest.

## Data Availability

Data sharing is not applicable to this article as no datasets were generated or analysed during the current study.
